# Reparative radiological changes of hip joint after TNF inhibitors in ankylosing spondylitis

**DOI:** 10.22088/cjim.9.3.303

**Published:** 2018

**Authors:** Kaouther Maatallah, Ines Mahmoud, Safa Belghali, Kawther ben Abdelghani, Olfa Saidane, Elyes Bouajina, Ahmad Laatar, Rawdha Tekaya, Leila Abdelmoula

**Affiliations:** 1 Department of Rheumatology, Kassab Institut Manouba 2010, El Manar Tunis University, Tunisia; 2 Department of Rheumatology Charles Nicolle Hospital, 1006 Bab Souika, El Manar Tunis University, Tunisia; 3 Department of Rheumatology, Farhat Hached Hospital, 4002 Medina of Sousse, Sousse University, Tunisia; 4 Department of Rheumatology, La Marsa Hospital, 2046 Sidi Daoud, El Manar, Tunis University, Tunisia

**Keywords:** Ankylosing Spondylitis, hip, imaging, TNF inhibitors

## Abstract

**Background:**

Hip involvement in ankylosing spondylitis (AS) is a common extraspinal arthritic manifestation, which is associated to a worse functional outcome. Little data are available on the effectiveness of conservative treatment strategies. The TNF inhibitors have been proven effective on AS activity parameters. Their structural effect on hip disease however, little is studied.

**Case presentation:**

We describe four new cases of reparative changes of a damaged hip joint after treatment with TNF inhibitors. The average of age was 32.5 (27- 36) years. There were 3 men and 1 woman. Hip involvement was bilateral in all cases. Etanercept was prescribed in 3 cases and infliximab in 1 case. At baseline, all patients had a painful and limited hip with high disease activity and an important functional impairment. After an average of 5.5 years of treatment with TNF inhibitors, the BASRI hip evaluated in antero-posterior x-rays of the pelvis remained unchanged at 2.4. The average of mean hip joint space was 2.9mm (2.3-3.6). A widening in hip joint space was observed in all cases with less subchondral cysts.

**Conclusion:**

TNF inhibitors seem to be effective on hip joint disease in patients with AS.

Hip involvement is a common extraspinal arthritic manifestation in patients with ankylosing spondylitis (AS) (1). In North Africa, AS occurs in 40% of patients after 10-year of disease duration (1). Hip replacement surgery in patient with end-stage hip disease remains the standard operation. Hip prosthesis has however a limited life span and is not devoid from complications (2). Little data are available on the effectiveness of conservative treatment strategies. TNF inhibitors have been proven effective on AS activity parameters. Their structural effect especially on hip disease is however little studied (2). We describe four new cases of reparative changes of a damaged hip joint after treatment with TNF inhibitors. Radiological hip progression was assessed using BASRI-h (bath ankylosing spondylitis radiographic index of the hip) and the average of hip joint space measured at three distinct sites according to Konsta et al. (3). 

## Case Presentation

There were 3 men and 1 woman. Hip involvement was bilateral in all cases. Etanercept was prescribed in 3 cases and infliximab in 1 case. Demographic, clinical, radiological and biologic data at baseline are presented in table 1. At baseline, all patients had a painful and limited hips with high disease activity (mean BASDAI (bath ankylosing spondylitis activity index) 4.7) (4-6), and an important functional impairment with a mean BASFI (bath ankylosing spondylitis functional index) at 6.6 (3.7-9), and a mean index of severity for osteoarthritis for the hip (ISH).at 15.6 (11-20) (ISH index entails  three main items which more or less include subjective issues like: pain or discomfort, maximum distance walking; and activities of daily living). 

**Table 1:** Demographic, clinical and radiographic characteristics of the four cases at baseline

	**Case 1**	**Case 2**	**Case 3**	**Case 4**
**Age (years)**	37	36	30	27
**Sex**	M	F	M	M
**Age at onset (years)**	23	28	19	17
**Delay in diagnosis (years)**	2	1	3	0.25
**Clinical Data**				
ISH	16.5	11	15	20
BASFI	9	5.8	3.7	7.6
BASDAI	6	4.6	4	4.25
**Biological and Radiological data**				
ESR,mean (mm)	44	15	43	78
CRP, mean( mg/l)	49	16	23	45
BASRI hip	2	3	2	2
Joint space, mean(mm)	2.7	2.2	2.8	2
AntiTNF alpha treatment	Etanercept	Infliximab	Etanercept	Etanercept
Delay between the first and the last radiograph	4	4	4	10

**ISH**: mean index of severity for osteoarthritis for the hip, **BASFI:** Bath Ankylosing Spondylitis Functional index, **BASDAI:** Bath Ankylosing Spondylitis Activity Index, **ESR:** Erythrocyte Sedimentation Rate, **CRP**: C Reactive Protein, **BASRI: **Bath Ankylosing Spondylitis Radiographic Index

**Figure 1:**
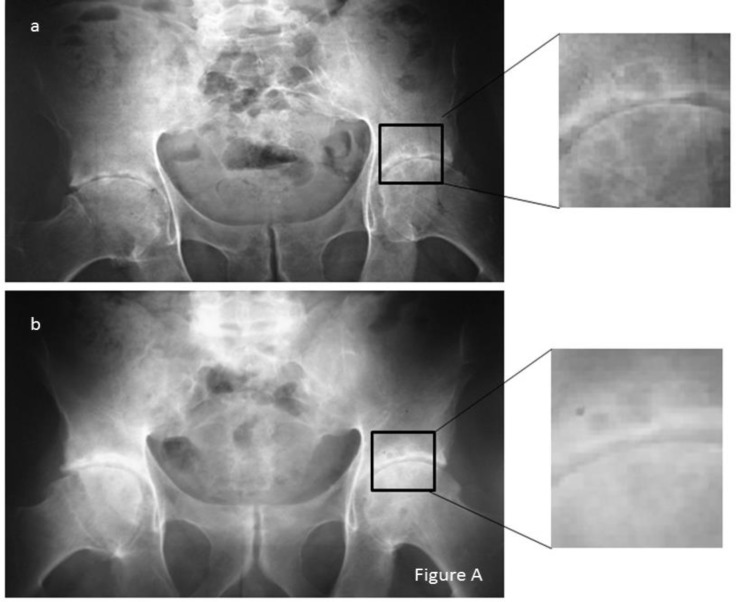
Radiographic images of anteroposterior x-rays of the pelvis in 2010 (a) and in 2014 (b) of case 1 showing reduction of the space narrowing with less subchondral cysts

After an average of 5.5 years of treatment with TNF inhibitors, analysis of antero-posterior x-rays of the pelvis showed a great improvement in disease activity with a BASDAI at 1.5 (0.5-2.8), in functional status with a mean BASFI at 1.6 (0.2-3.3) and a mean ISH at 2.5 (0-8). The BASRI hip evaluated in antero-posterior x-rays of the pelvis performed in same circumstances remained unchanged at 2.4. The average of mean hip joint space was 2.9mm (2.3-3.6). A widening in hip joint space was observed in all cases with less subchondral cysts (Fig1).

## Discussion

Because of the central function of the hip joint, impairment of hip functioning is associated to a worse functional outcome and may seriously worsen prognosis of the disease in patients who are usually active and young (4). Short-term follow-up studies have suggested a radiographic inhibition of the hip under TNF inhibitors (5-7). The long-term data reported by Konsta et al. have strengthened this hypothesis (3). 

Our data, as well as those of literature, suggest that the positive effect of TNF inhibitors observed on structural changes in patients with rheumatoid arthritis (RA) is also seen on osteodestructive changes in patients with AS. In fact, unlike the classical structural changes of AS in the spine, inflammation of the subchondral bone marrow in the hip (8), leads to an erosive disease with joint space narrowing which causes joint destruction. Olthough, there is not a new bone formation. A similar case of hip radiographic repair after a year of infliximab treatment in a patient suffering from SA has been reported in the literature (9). 

Radiographic repair of the hip in isolated RA cases have also been reported (10-13) including four patients with juvenile-onset RA. The mechanisms of improved hip joint space have not been studied. Nonetheless, it has a case reported by Sakuma et al (13) where it was investigated if hip radiographic repair reflected a true histopathological repair on the femoral head. This was a juvenile-onset RA patient with radiographic improvement in the hip under TNF inhibitor. This study showed an eburnated femoral surface and partially coated by fibrous tissue. This tissue was identified microscopically as fibrocartilage. These findings suggest that the x-ray repair could be due to the presence of fibrous cartilage overlying a partially repaired joint 

In conclusion, TNF inhibitors seem to be effective on hip joint disease in patients with AS. Further studies are however required to confirm and evaluate their effect on preventing hip impairment.

## Conflict of Interest:

There is no conflict of interest to declare
